# Circadian dysfunction and cardio-metabolic disorders in humans

**DOI:** 10.3389/fendo.2024.1328139

**Published:** 2024-04-29

**Authors:** Natalia Marhefkova, Martin Sládek, Alena Sumová, Michal Dubsky

**Affiliations:** ^1^ Diabetes Centre, Institute for Clinical and Experimental Medicine, Prague, Czechia; ^2^ First Faculty of Medicine, Charles University, Prague, Czechia; ^3^ Institute of Physiology, The Czech Academy of Sciences, Prague, Czechia

**Keywords:** circadian clock, circadian rhythm disruption, cardiovascular disease risk, type 2 diabetes mellitus, insulin sensitivity, glucose tolerance, time restricted eating

## Abstract

The topic of human circadian rhythms is not only attracting the attention of clinical researchers from various fields but also sparking a growing public interest. The circadian system comprises the central clock, located in the suprachiasmatic nucleus of the hypothalamus, and the peripheral clocks in various tissues that are interconnected; together they coordinate many daily activities, including sleep and wakefulness, physical activity, food intake, glucose sensitivity and cardiovascular functions. Disruption of circadian regulation seems to be associated with metabolic disorders (particularly impaired glucose tolerance) and cardiovascular disease. Previous clinical trials revealed that disturbance of the circadian system, specifically due to shift work, is associated with an increased risk of type 2 diabetes mellitus. This review is intended to provide clinicians who wish to implement knowledge of circadian disruption in diagnosis and strategies to avoid cardio-metabolic disease with a general overview of this topic.

## 1 Circadian clock and its parameters

Circadian rhythms are driven by endogenous cellular clocks that are responsible for temporal programming of physiological and behavioral processes, as well as the synchronization of these processes with changes in environmental conditions within a 24-hour cycle. These clocks generate rhythm with an approximate 24-hour period owing to a molecular transcriptional-translational feedback loop (TTFL) composed of families of clock genes (e.g. human genes PER1-3, CRY1-2, NR1D1-2, RORA-C, BMAL1-2, CLOCK, NPAS2) that are quite kept across different animal phyla (1). The encoded proteins function as transcriptional activators or repressors, controlling the expression of their partners and downstream clock-controlled genes, which govern tissue-specific rhythmic processes. Levels of regulation in addition to TTFL such as phosphorylation of key proteins, further guarantee the stability, precision and temperature compensation of the cellular clock (2).

**Figure 1:**
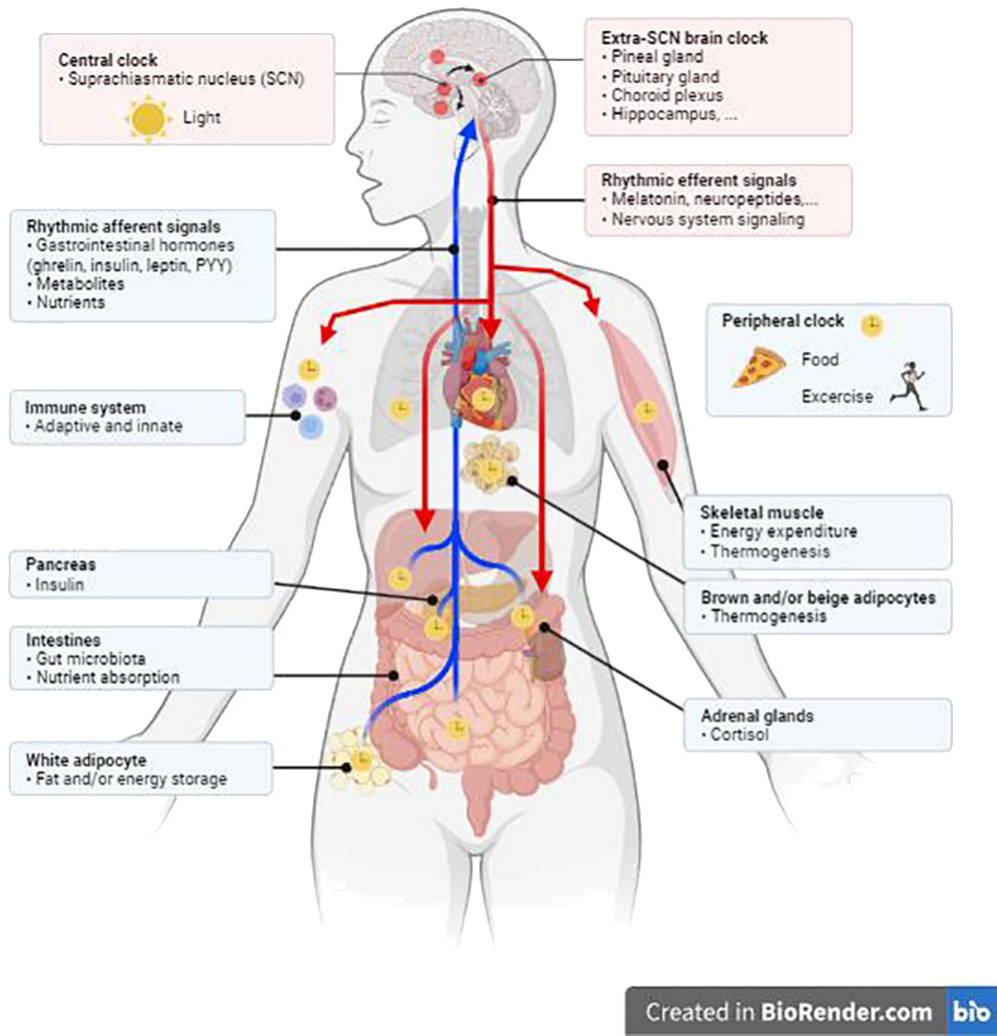
The peripheral circadian clock is regulated by the central clock in the suprachiasmatic nucleus (SCN) through responses to hormones, the neurological system, physical activity, and eating habits. Created in BioRender.

In humans, as in other mammals, the clocks are mutually interconnected to form a hierarchical system, which is governed centrally from a structure in the hypothalamus called the suprachiasmatic nucleus (SCN) (3). These paired nuclei are morphologically and functionally arranged as the main trigger of rhythmicity at the systemic level (**Figure 1**) and receive all signals directly from specific cells in the retina, which enable synchronization of the physiological biorhythm with light-dark cycles. Other internal clocks in the brain and elsewhere in the body use some of the SCN-controlled rhythmic signals, in addition to external time, to synchronize with each other. This is achieved via multiple signals which include daily changes in the tonus of autonomous nerves system, hormone levels, body temperature, metabolic state, etc. Although the SCN clock receives feedback from peripheral tissues, it is predominantly synchronized by the light/dark cycle and is highly resistant to most of the signals under standard conditions of energy balance (4).

Signals which entrain circadian clocks are called “Zeitgeber” (time giver or time cue in German), a term which was first used by Jürgen Aschoff, one of the founders of the field of human chronobiology (5). His work demonstrated the existence of endogenous (internal) biological clocks in humans maintained in time-isolation. In addition, he showed that certain exogenous (external) cues, which he called Zeitgebers, influence the phase and period of these internal clocks (5).

### 1.1 Sleep parameters and their evaluation

Sleep is the most important factor when setting one’s circadian rhythmicity. Its timing is controlled by a complex process that requires coordination between the circadian clock in the SCN and hormonal homeostasis (6). Nevertheless, other factors such as mental and physical health can also be very important aspects for quality of sleep.

Most of the latest sleep studies have focused on basic sleep parameters that include sleep duration (e.g., insomnia, hypersomnia), sleep quality (e.g., fragmentation) and sleep timing (e.g., delayed, advanced, irregular, non-24 hour) (7). These sleep characteristics are often related to individual chronotype (6), as is discussed below.

There are multiple approaches (both subjective and objective) to evaluating sleep parameters, depending on the type of the study, experimental conditions and expected outputs (8).

A subjective evaluation of sleep quality is attained by using specialized self-reported questionnaires. The most commonly used questionnaires include, but are not limited to, the Pittsburgh Sleep Quality Index (PSQI), the Jenkins Sleep Scale (JSS), the Leeds Sleep Evaluation Questionnaire (LSEQ), the Insomnia Severity Index (ISI), and the Epworth Sleepiness Scale (ESS) (**Table 1**). Some questionnaires include additional questions regarding socio-economic status in order to collect information on social deprivation (EPICES) or employment conditions (KARASEK) (7).

**Table 1:** Types of questionnaires used to assess sleep quality.

Name of questionnaire	Number of questions	Result score range	Focus/output
*Pittsburgh Sleep Quality Index (**PSQI***	7	0-21	7 components to asses overall sleep quality >5 considered as a significant sleep disturbance
*Insomnia severity index **(ISI)***	17	0-28	evaluates symptoms of insomnia, valuation of sleep difficulty intensity >15 score indicates moderate to severe insomnia, 8- 14 subthreshold insomnia
*Morningness –Eveningness Questionnaire **(MEQ)***	19	16-86	psychological behavior 5 chronotypes (extreme morning, moderate morning, intermediate, moderate evening, extreme evening)
*Munich Chronotype Questionnaire **(MCTQ)***	17	Sleep time in hours	primarily focused on sleep timing relation to age, gender and self-declared body mass index MSFsc: midpoint of sleep on work-free days, corrected for sleep-debt to asses chronotype
*Sleep Timing Questionnaire **(STQ)***	18	Direct responses	used to determine habitual bedtime and wake times

The PSQI is a self-report questionnaire, developed by researchers at the University of Pittsburgh (8), that evaluates sleep quality over a period of 1 month. The evaluation consists of 19 individual items, which form 7 components that provide an overall score. It is the most commonly used subjective assessment of sleep quality and is therefore utilized as a standardized sleep questionnaire for clinicians and researchers. When assessing the PSQI, seven component scores are evaluated, each receiving a score from 0 (no difficulty) to 3 (severe difficulty). The component scores are added together to give an overall score (range 0 to 21). Higher scores indicate poorer sleep quality. The PSQI is a general assessment and includes subscales that measure total sleep time, sleep onset latency, sleep efficiency (ratio of total sleep time to time spent in bed), sleep disturbances, degree of fragmentation (i.e., the number of arousals in relation to total sleep time), use of sleep medications, daytime alertness and total waking time. Little is currently known about how the various constructs, which comprise the PSQI, are individually related to diabetes control (9).

The Sleep Regularity Index (SRI) is a relatively new metric for measuring sleep regularity. The SRI evaluates the probability (presented as a percentage) of an individual being in the same state (awake or asleep) at any two time points 24 hours apart (10). Delays in circadian sleep/wake cycles and unfavorable cardiometabolic (CM) outcomes, such as an increased 10-year risk of cardiovascular disease, obesity, hypertension, T2DM markers, high fasting blood glucose levels and glycated hemoglobin (HbA1c), have been linked to lower SRI scores (11). These metrics, implemented as supplementary techniques used to characterize sleep regularity in ongoing studies, may provide a better understanding of the association between sleep and cardio-metabolic disorders.

**Figure 2:**
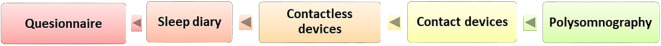
Comparison of sleep assessment methods by levels of accuracy.

Essentially, there are objective and subjective methods to classify sleep parameters in a patient. With regards to the objective assessment, polysomnography (PSG) is considered the gold standard when evaluating sleep physiology (12). This method implements a significant amount of complementary information that can be useful in various ways, such as in diagnosing sleep disorders (**Figure 2**). Polysomnography requires overnight monitoring of the patient in a specialized medical facility, and it is therefore not suitable for the assessment of sleep parameters in real life conditions. Various devices for monitoring behavioral activity (actigraphy) and other sleep parameters have been developed for this type of “field study.”

Actigraphy is used to evaluate activity and rest cycles to determine sleep parameters such as timing, duration and fragmentation. Behavioral activity is monitored by a small motion sensor detector (accelerometer) worn like a watch on the non-dominant wrist (13). It enables the tracking of sleep over extended periods of time in a non-laboratory environment. These devices allow long-term non-invasive examination of circadian rhythm and sleep disruption in patients with various disorders, including neurodegenerative (14) and CM disorders (15).

The new wave of fitness trackers and other health-optimizing (‘biohacking’) gadgets is booming, multisensory devices are becoming popular due to the fact that they are labelled ‘user-friendly’ by the trade industry. These devices are capable of receiving a wide range of biosignals from their users. However, the effectiveness of these commercial devices remains controversial and their reliability has yet to be tested. In a recent study, four wearable (*Fatigue Science Readiband, Fitbit Alta HR, Garmin Fenix 5S, Garmin Vivosmart 3)* and three non-wearable (*EarlySense Live, ResMed S+, SleepScore Max)* consumer sleep-tracking devices were tested for performance in thirty-four healthy young adults (22 women; mean age 28.1 ± 3.9 years). All sleep data from these devices were compared with the data from actigraphy and PSQ. Most devices performed on par with (in some cases even outperforming) actigraphy in measuring sleep-wake performance, while the Garmin devices fared worse (16). A Korean study compared another well-known activity tracker called Fitbit Charge HR to actigraphy in 16 healthy young adults, and the results showed high accuracy of Fitbit tracker in assessing sleep and measuring circadian rest-activity rhythm (17). Ring-sized wearables are increasingly used by many consumers worldwide. One study tested the ŌURA ring and compared its performance in measuring sleep and sleep stages with that of the PSG. Sleep was monitored during a single laboratory night in 41 healthy adolescents and young adults (13 females; mean age: 17.2 ± 2.4 years) (12). In our opinion, this well-designed study provided promising results that confirm the performance of this device. The study showed that the summary variables for key sleep parameters, such as sleep onset latency, total sleep time, and waking after falling asleep, did not differ between the ŌURA ring and the PSG. The ŌURA ring was 96% in accordance with the PSG in detecting sleep, other parameters like wakefulness (48%), light sleep (65%), deep sleep (51%) and REM sleep (61%) were less in conformity. However, the ŌURA ring produced considerable variability in measuring sleep depth (underestimation) and REM sleep (overestimation) (18, 19).

These results suggest that many commercial sleep monitors show promising performance in monitoring sleep and wakefulness. In future studies, they should be tested under different conditions (different populations and environments) in order to further investigate their broader validity and applicability in medical research as an alternative tool to actigraphy for sleep assessment and circadian rest-activity rhythm measurement in a real-world environment. The increasing availability of more sophisticated devices, which go beyond mere activity recording, could provide clinicians with the opportunity to analyze diversity of sleep and physiological events during sleep in more detail.

### 1.2 Chronotype and its evaluation

Individuals differ greatly in their preferences for the time of day at which they perform certain activities and when they sleep. This phenomenon is called a chronotype (20) and is the natural preference of the body for wakefulness and sleep relative to solar time (and social time). Among the general population, chronotypes exhibit almost a normal Gaussian distribution (21).

It is assumed that the chronotype is determined by the central circadian clock and is also expressed via a peripheral clock within an organism (22). The exact mechanism underlying the chronotype is not yet fully understood. It is most likely related to the duration of the endogenous period of the SCN clock, since individuals with clocks that run with longer periods tend to be later chronotypes (22). However, other clock parameters may be involved, such as amplitude and its ability to entrain with actual light exposure (23). In addition, genetic, social, and environmental factors can also affect he chronotype (24). A chronotype changes considerably over the course of a lifetime, with adolescents being late chronotypes, while children and older people tend to be early chronotypes (25). A chronotype also appears to be dependent on biological sex since males tend to be late chronotypes more often than females (23), however, this difference is age-dependent (26). The factors that determine an individual’s chronotype are therefore complex.

A chronotype can be determined subjectively using standardized, validated self-assessment questionnaires, as well as objectively by recording daily behavior using the methods described above or by analyzing biochemical/molecular biomarkers in non-invasively collected biological samples. The latter approach can provide precise information about the actual phase or period of the internal circadian clock.

There are 2 commonly used chronotype questionnaires for subjective assessment: the Morningness - Eveningness Questionnaire (MEQ), which assesses the preferred timing of various behaviors and the Munich Chronotype Questionnaire (MCTQ), which takes into account differences in sleeping patterns between workdays and work-free days during the week (21). The MEQ questionnaire includes 19 specific questions to determine whether a person’s circadian rhythm peaks (in terms of alertness) in the morning, evening or in between these two periods. Most of the questions are preferential, e.g., the respondent is asked to indicate when they would like to wake or sleep if they had a choice (full control over their sleep/wake cycle). The MEQ questionnaire categorizes groups into morning types, evening types and intermediate types. The score ranges from 16 to 86, with lower scores indicating the evening types. The MCTQ is a useful tool in assessing chronotypes based on self-reported times of sleep or wakefulness, as well as sleep latency and inertia. The questionnaire expresses the chronotype as a midpoint of sleep on work-free days (Mid-Sleep on Free Days or MSF). The MCTQ and its significantly shortened version, µMCTQ (27), have been proven useful in assessing chronotypes in studies involving large populations, primarily through the use of an online version of the questionnaire (21). A more demanding approach is to identify a chronotype by incorporating the MCTQ into a group of questions within a population-representative sociodemographic survey. These are carried out by in-person visits to households, which provide data on chronotypes and their correlation with various social, health and life-style factors (20).

Methods used to estimate a chronotype objectively are mostly based on wrist actigraphy, i.e., the same methodology mentioned above for the assessment of sleep timing. One modification of this approach is to measure circadian rhythm parameters by monitoring wrist temperature, which is partially subject to regulation by the circadian system (28). Newer devices are equipped with a temperature sensor that is attached to the inside of the wrist with medical tape, with the sensor surface placed over the radial artery of the non-dominant hand. This temperature marker has been validated to reflect the circadian rhythm, which is related to the timing of light exposure and the amplitude of melatonin secretion.

Melatonin is produced in the pineal gland and regulated mainly by the SCN clock and light exposure (29–31). Therefore, an increase in melatonin levels exceeding the low diurnal levels found under low light conditions is used as a marker for the endogenous clock and determines the onset of subjective night, which varies according to chronotype (14).

More recently, other non-direct light-responsive markers have been introduced. They are mostly based on the molecular mechanism underlying circadian rhythmicity at the cellular level, and they have the ability to detect the phase of the molecular clock in human samples, such as blood, skin, oral mucosa, and hair follicles (22).

### 1.3 A chronotype and its association with CM disorders

All the approaches mentioned above require collecting samples from subjects in short intervals around the clock. As a result, there has been an effort to introduce a technology that can reliably detect the phase of the clock from a single sample. Novel technologies suitable for rapid assessment of the phase of the circadian system and chronotype in outpatient care would provide useful diagnostic information in treating not only sleep disorders but also lifestyle-related disorders. Indeed, having an extreme chronotype is recognized as one of the risk factors or subclinical predictors of metabolic and cardiovascular disorder (CVD). Evening (late) chronotypes are generally more susceptible to CVD than are morning chronotypes (32). The pathomechanisms are unclear, but they are often explained as being related to either lifestyle factors (e.g., smoking and drinking alcohol; both more common in late chronotypes) or an increased susceptibility to disruption of rhythms (see below). For example, late chronotypes have significantly higher fasting blood glucose, HbA1c, triglycerides and low-density lipoprotein cholesterol (LDL) than their morning counterparts; however, no significant differences were found in BMI, the energy intake or blood pressure (BP) (33). When gender was involved as a variable in one study, a late chronotype correlated with a higher BMI, specifically in women (20). An extremely late chronotype, normalized for age and sex, has significantly lower HDL levels and a higher LDL/HDL ratio than those of an extremely early chronotype (20). A late chronotype is also positively correlated with levels of proteins associated with insulin resistance and cardiovascular disease, specifically retinoic acid receptor protein 2, fatty acid-binding protein adipocytes, tissue-type plasminogen activator, and plasminogen activator inhibitor (32).

Many studies have repeatedly confirmed that the risk of developing type 2 diabetes mellitus (T2DM) is higher in late chronotypes (34). Studies revealed that a chronotype could have a significant impact on insulin resistance since late chronotypes are associated with less favorable glycemic control; these studies did not take sleep duration or overall physical activity (PA) into account (35, 36).

Interestingly, recent evidence suggests that not only late chronotypes but also extremely early chronotypes may be associated with increased CVD markers such as lower HDL, higher triglycerides and an increased atherogenic plasma index (26). Due to a lack of evidence, we can currently only speculate about the underlying mechanisms. Since extreme chronotypes tend to have a larger phase angle between their endogenous clock and external time than do non-extreme chronotypes, the amplitude of their rhythms may be negatively affected. This may be further exacerbated by weak entrainment cues in modern urban settings. Instead of continually adapting to external time, extreme early chronotypes may experience sudden phase shifts when their clock runs out of sync with the light-dark cycle. This may destabilize the endogenous rhythm governing the metabolism of fatty acids and cholesterol.

Having a late chronotype is a strong predictor for a higher discrepancy between social time and endogenous (biological) time, which is referred to as social jet lag (21). This discrepancy is common in modern society and can be a result of either poor synchronization of the preferred sleep-wake cycle with social time (by choice or due to illness) or night shift work schedules. Some evidence indicates that the mismatch between preferred sleep time and long-term work schedules is associated with an increased risk of T2DM due to the disruption of glucose metabolism and a reduction in glucose tolerance (37). This mismatch could worsen disease prognosis in patients already diagnosed with T2DM (37). However, social jet lag is not only limited to shift workers since a significant portion of the population with standard work schedules experience this condition to a certain degree. In a recent population-representative study, 1957 blood samples were analyzed for 9 different biomarkers and results revealed significant associations between sleep phase preference, social jet lag and CVD biomarkers (26).

There are, however, other non-circadian factors that are linked to poor health and increased CVD risk; late chronotypes are more associated with unhealthy dietary habits (e.g., late-night eating), reduced PA and/or a low-quality social life (20). Therefore, the relation between late chronotypes, social jet lag and the risk of CVD may involve both environmental and behavioral factors.

### 1.4 Sex differences in sleep, chronotype and their relationship with CM disorders

Women are generally underrepresented in many research studies focusing on human physiology, with circadian and sleep research being no exception. Many human studies tend to exclude women from participation due to fluctuation in female hormone levels combined with the overall neuroendocrine system would possibly modulate circadian responses. A British study on the association between sleep and cognitive performance in men and women found that some circadian characteristics, such as the natural oscillation of the circadian clock and the amplitude of the melatonin rhythm, differ between men and women; however, no differences were observed with regards to usual amount of time spent in bed, sleep duration, or sleep quality as measured by the PSQI. Interestingly, the study revealed differences between the sexes with respect to circadian rhythmicity in cognitive skills; women experienced greater night-time impairment in cognitive performance than did men (38).

Sex-related sleep disturbances may impact CM functions via disruption of circadian regulation as well as downregulation of the metabolic pathways. The SWAN (Study of Women’s Health Across the Nation) cohort study in perimenopausal women (mean age: 51 years) discovered that the greater the variability in bedtime, the higher BMI, higher body fat percentage, and lower lean mass percentage (39). As mentioned earlier, BMI is positively correlated with a late chronotype in women, but no such correlation is evident in men (21). Furthermore, social jet lag is significantly associated with higher cholesterol levels in the younger female cohort, but not in the male cohort. Based on a composite of blood pressure, fasting blood glucose levels, lipid levels, and waist circumference measurement, another study conducted on female hospital workers revealed that women who worked rotating shift schedules had higher CM risk scores (40).

It is becoming more evident that physiological (hormonal levels), cognitive (spatial processing, emotional condition, and linguistic fluency) and social factors (family and childcare responsibilities) interact to create a landscape of different vulnerabilities to circadian disruption in men and women (41). However, in addition to the circadian phenotypes, differences between the sexes with regards to sleep and CM functions may be mediated independently of, or downstream from, circadian processes, i.e., at the level of hypothalamic-pituitary-adrenal axis function and fluctuations in reproductive hormones (38), which are linked to a range of sleep problems and particular sleep disturbances, including insomnia or breathing issues throughout the course of various phases of reproductive aging (42).

### 1.5 What can we learn from a UK biobank study on sleep and their connection to T2DM risk?

The majority of research focusing on the connection between circadian disruptions, sleep, and T2DM risk evaluates each sleep parameter separately rather than as a composite. To address this oversight, scientists analyzed data from a sizable biomedical database and research resource that included detailed health and genetic information from half a million UK participants between 2006 and 2010 and involved over 500 000 participants nationwide (43). After nearly nine years of follow-up, this extensive population-based cohort study on the UK Biobank was complete. A total of 6,940 case subjects with incident T2DM had been documented. The information was utilized to assess the correlation between sleep factors, genetic risk, and their combined effect on incidence of T2DM (37, 44). After an average of 8.5 years of follow-up CVD risk assessment, analysis revealed that early chronotypes were linked to a lower risk of coronary heart disease (33). High serum 25-hydroxyvitamin D concentrations are linked to a lower risk of T2DM, and these correlations are negatively influenced by sleep patterns, with daytime sleepiness (excessive sleepiness or hypersomnia) being the main contributor. To address this oversight, scientists analyzed data from a sizable biomedical database (45). The results of another large prospective population-based cohort study were published with the aim of promoting healthy sleep and circadian patterns throughout the population.

Another clinical observational study (6)used well-established sleep parameters (including long or short sleep duration, sleep scores, snoring, late chronotype, excessive daytime sleepiness and insomnia) to categorize sleep quality and circadian pattern as unfavorable, intermediate, or favorable with regards to the development of T2DM; the study involved 360 403 participants and 9 years of follow-up. Each participant was placed into a category after submitting a self-reported questionnaire. The following criteria were also established for each category in another multivariable-adjusted model: age, sex, education, socioeconomic status, PA level, smoking status, alcohol consumption, BMI, CVD, cancer, hypertension, and family history of diabetes. A genome-wide association study used genotyping to evaluate genetic data and categorize the polygenic risk score as low, intermediate, or high risk (46). The incidence of T2DM was more than twice as high (5.53%) in the group of participants with high genetic risk as it was in the group with a low genetic risk (2.01%). Even after accounting for different sleep factors, the association between genetic risk and T2DM incidence remained constant, indicating that sleep, circadian rhythms, and genetic risk were all independently linked to the incidence of T2DM. It is important to mention that this study enrolled only individuals of European ancestry; therefore, the results cannot be generalized for all ethnic groups since racial differences were not been taken into account. Another limitation of this study is its omission of other scientifically confirmed factors (e.g., shift work, late-night eating, low PA), which contribute to poor sleep patterns and, as a result, a possible increase in the risk of developing T2DM.

## 2 Impact of circadian disruption on cardiovascular and metabolic functions

**Figure 3:**
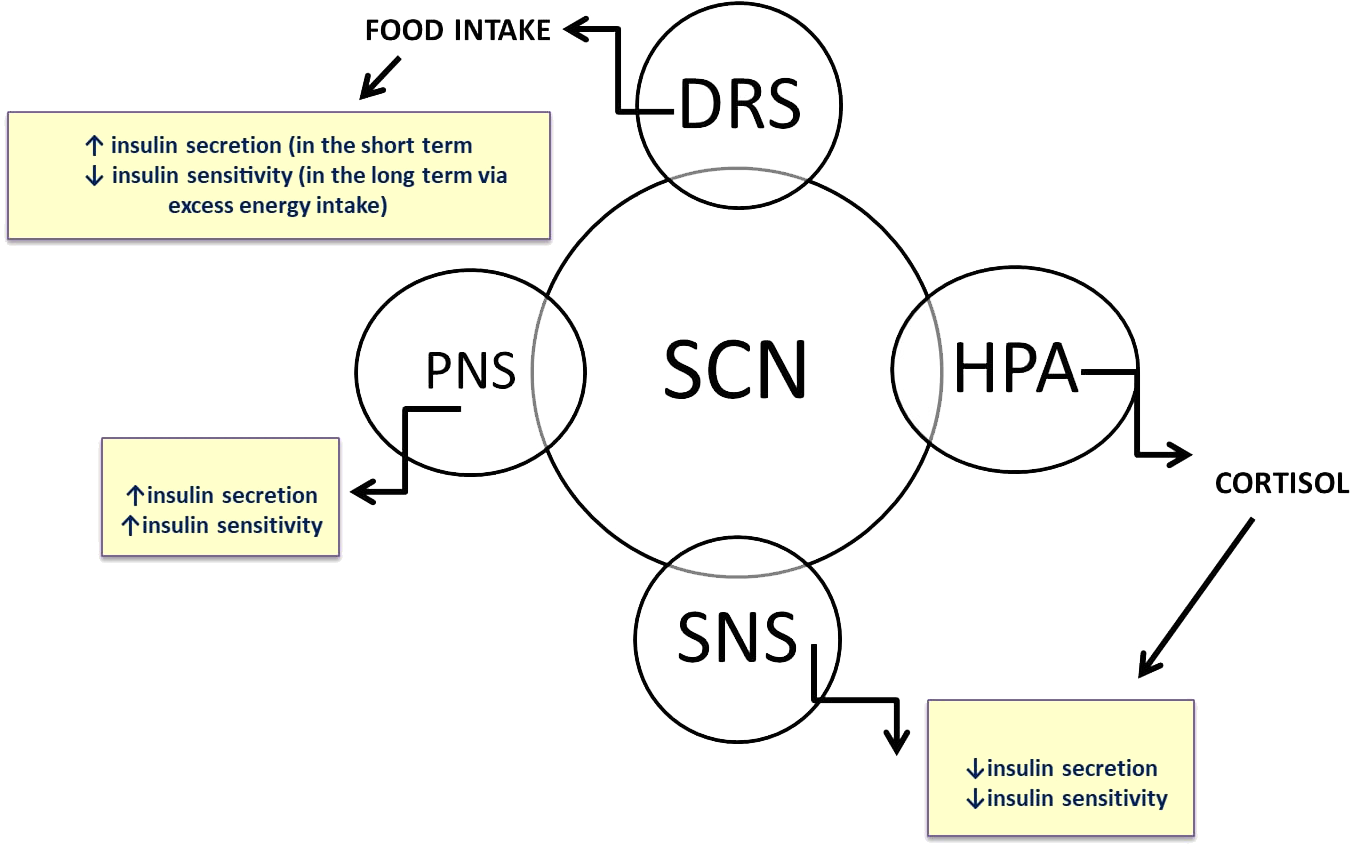
The central clock, located in the suprachiasmatic nuclei of the hypothalamus (SCN), synchronizes clocks in peripheral organs through various neuronal and humoral pathways. Glucose tolerance is directly regulated by circadian rhythm via the neuroendocrine system.

An individual’s health is dependent on the synchronization of all of the internal clocks in the body, as well as on the synchronization of said clocks with the external environment. The circadian system, which regulates metabolism and heart function, ensures that associated organs can perform at their best to meet the expected demands of the daytime and nighttime hours. Recent research on SCN-lesioned rodents revealed that the SCN clock regulates the diurnal rhythm in whole-body insulin sensitivity (IS), and a wealth of evidence suggests that the human circadian system governs the metabolism of glucose, lipids, and energy (47). The SCN clock regulates the release of hormones that impact glucose tolerance, such as cortisol, growth hormone, and melatonin (**Figure 3**). Muscle tissue exhibits a diurnal rhythm with higher IS in the morning than in the evening (48). However, cardio-metabolic functions are also modulated by lifestyle factors, such as PA, meal timing and sleep patterns. Food intake serves as a strong timing signal to certain peripheral clocks, while nighttime light exposure is the primary disruptor of the central clock in the SCN (49). Consequently, eating at the “wrong” time of day could disrupt the synchronization of clocks in different tissues. An imbalance between these variables and an individual’s circadian rhythm may therefore increase the chance of developing metabolic and cardiovascular diseases or conditions, primarily T2DM, obesity, insulin resistance, metabolic syndrome, dyslipidemia, or high blood pressure (BP) (33). The fact that the risk of adverse CVD events varies according to the time of day, peaking at 9:00 AM and then again in the evening at 8:00 PM, suggests that circadian disruption and pathology work together (50). A recent study involving 91 adults with obesity and prediabetes evaluated *post hoc* associations between CM risk factors, physical activity (PA), and circadian rhythm parameters (monitored by continuous wrist-temperature measurements) (51). The results showed that a more consistent circadian rhythm was associated with lower CVD risk. The relationship of PA to either cardio-metabolic risk or circadian rhythm had no effect on the incidence of CVD. Physical activity (PA) was only linked to greater circadian stability in individuals with lower systolic blood pressure (SBP). The results are extremely encouraging for future research or clinical practice in this area.

Several lines of evidence have shown that the molecular clock plays a role in lipid metabolism. Nocturnin, a gene expressed in a circadian manner and one which produces an enzyme with deadenylase activity (52, 53), is known to have a key role in the regulation of lipid metabolism. The circadian clock is also influenced by excessive fat intake and metabolic changes. Mice fed a high-fat diet exhibited symptoms of metabolic syndrome, including hyperglycemia, hyperlipidemia, and obesity, which are all likely due to widespread reprogramming of the circadian clock, as well as the transcriptome and metabolome (54). Further research is needed to understand how fat accumulation and metabolic disease can disrupt the circadian clock (55–57).

In the following text we discuss lifestyle factors, such as sleep patterns, physical activity, and food intake that lead to circadian misalignment and may increase the risk of cardiovascular and metabolic disorders.

SNS, sympathetic nervous system; PNS, parasympathetic nervous system; HPA, hypothalamic-pituitary-adrenal axis; DRS, dopaminergic reward system.

Currently, studies are on rise that aim to better characterize how certain unhealthy lifestyle factors (namely poor sleeping habits, unfavorable eating schedules and insufficient PA) can negatively influence glycemic control in patients with T2DM, obesity and an elevated risk of developing CVD (51, 58, 59).

### 2.1 Sleep disruption and CVD risk

Sleep duration is a significant risk factor for developing diabetes. Research has shown that people who regularly sleep for either very short or very long periods of time (based on the “optimal” sleep duration of 7 to 8 hours) are more likely to develop T2DM (48). However, other factors, such as an undiagnosed illness, may contribute to the detrimental effects that extended sleep duration has on health. One explanation for how sleep disturbance and deprivation affect IS is that they bring about a change in the sympathovagal balance (60).

In patients with diabetes mellitus, optimal blood glucose control is assessed on the basis of specific parameters like glycemic variability (GV), which refers to fluctuations in blood glucose levels over a specific time interval; these measurements are presented as time in range (TIR, glycaemia from 3.9 to 10 mmol/L), time below range (TBR) or time above range (TAR). In a recent study (61), 28 T2DM patients treated with continuous subcutaneous insulin infusion therapy had their glycemic parameters and sleep duration monitored, and results indicated that PA and longer sleep durations were positively associated with lower daily GV. It is interesting to note that sleeping for an extra hour could reduce GV by 0.72% (61). The results suggest that prolonging sleep duration in T2DM patients can significantly improve diabetes control, especially with respect to preventing peripheral neuropathy.

The effect that disruptions in sleep patterns have on glucose tolerance was previously associated with the hormone melatonin; this conclusion was reached after the discovery that genetic variations in MTNR1B – the melatonin receptor gene – correlate with impaired fasting glucose in T2DM patients (30). However, the effects of melatonin and variations in MTNR1B on metabolism are highly contradictory, and the role of melatonin in the pathophysiology of glucose tolerance remains controversial (30, 48, 59). Elucidating the benefits or detriments of melatonin is crucial for the development of melatonin agonist/antagonist drugs (30).

### 2.2 Timing of PA and CM risk

Physical activity is another important factor in maintaining healthy CM functions. A moderately to vigorously intense level of PA is recommended by the World Health Organization in order to prevent CVD. Between 1987 and 2012, one US research study tracked 5807 men and 7252 women aged 45 to 64 years (all participants were initially free of CVD), and the results indicated an inverse correlation between PA and CVD (58). Physical activity was the most important factor to correlate with circadian rhythm parameters in healthy young men with various BMIs (optimal, fair and poor). These men were monitored daily for changes in their wrist temperature as a measure of circadian rhythm. Other parameters were also observed, such as body composition, cardiorespiratory fitness, actigraphy, daily nutritional and sleep habits, as well as fasting lipid, insulin and glucose levels (62).

In the above-mentioned study involving T2DM patients, PA carried out at greater than 1.5 Metabolic Equivalents of Task or METs* for at least 1 hour was associated with lower GV on that given day; this was the case even though overall PA levels remained low (61). Furthermore, low bolus insulin doses were associated with higher GV, which could be due to insufficient doses of insulin at mealtimes or the absence of bolus insulin when snacking. Similarly, it is understandable that overall glucose metrics are higher during sleep than during wakefulness because of the longer intervals between meals during sleep (61). Another recent study in patients with T2DM showed that proper PA timing, based on internal time (chronotype), can help to regulate impaired glucose metabolism (63). These findings demonstrated that exercise performed at random times of the day was less effective than workouts carried out in the morning and evening for patients with early and late chronotypes, respectively. Results indicated improvement in levels of HbA1C, fasting blood glucose, triglycerides, HDL, LDL, total cholesterol, as well as an overall improvement in quality of life in people with T2DM.

There should be more widespread awareness of the importance of regular PA combined with sufficient sleep and healthy eating habits for T2DM patients. It is unclear whether developing exercise programs tailored to a specific chronotype could help people with T2DM manage their condition. Further research is necessary to determine how increasing the level and timing of PA can affect circadian system status in different populations.

The skeletal muscle circadian clock establishes strong rhythms during the oxidative metabolism in the tissue and these rhythms peak in the evening (64). It is therefore tempting to hypothesize that a decline in metabolic health is partially caused by disruptions in the muscle tissue rhythms, which are linked to circadian misalignment. Therefore, decreased oxidation in skeletal muscle may also be linked to the onset of T2DM (65).

### 2.3 Meal timing and CM risk

Dietary recommendations have recently focused more on meal timing rather than on mere meal quantity and quality (66). The field of chrono-nutrition is a relatively new area of study that examines the relationship between the circadian system and food intake.

Food consumption ensures that peripheral clock timing is in sync with the day/night cycle. The timing of food intake drives rhythmic processes in the metabolic organs. This is significant because some metabolic hormones exhibit daily variations due to the SCN clock, which is unaffected by food intake under energy-balanced conditions. Several hormones have been shown to have daily oscillations, the best known of which are melatonin, cortisol, gonadal steroids, prolactin, thyroid hormone, and growth hormone (GH). The so-called nutrient-sensitive hormones, which include insulin, leptin, ghrelin, and adiponectin, also oscillate on a circadian basis, and their release is influenced by environmental factors such as feeding time and light-dark cycles (31).

The hormone cortisol, which controls energy levels and primes the body for an active phase, is released in anticipation of awakening and peaks in the morning hours (7 a.m. – 9 a.m.) an individual with a well-synchronized SCN clock. Among all glucocorticoid hormones, cortisol is one of the most widely-studied from a circadian point of view (31). In one study, jet lag and sleep desynchronization were shown to increase cortisol levels in humans (67), and elevated cortisol has been associated with several pathologies, including cardiometabolic disease and sleep disorders (68, 69).

Insulin and ghrelin are two important metabolic regulators, and several circadian factors are now known to influence their secretion and activity. In another study, shift work has been shown to contribute to a rise in insulin secretion and a decrease in insulin sensitivity, potentially implying a pre-diabetic condition (31). One study revealed that circadian misalignment induced by sleep deprivation increased markers of insulin resistance and inflammation (70). The main role of the hormone ghrelin is appetite stimulation. One study involving shift workers revealed that their normal ghrelin cycle becomes disrupted, which may explain why overeating is so common among such workers (71).

On average, a person eats three meals a day (one 8am, one at 1pm, and one at 6pm), and ghrelin levels peak just before these mealtimes (72). In particular, eating breakfast on a regular basis can help regulate plasma lipid levels and glucose homeostasis, but it can also act as a morning clock synchronization cue (66). However, late-night meals are linked to inadequate glycemic control in individuals with T2DM (73). In a recent study, two separate 56-hour sessions of a random crossover design were used to monitor the metabolism of older subjects in a whole-room respiratory chamber. The findings demonstrated that maintaining lipid oxidation requires eating breakfast and avoiding late-night meals. These finding suggest that human oxidation, or storage of ingested food, is influenced by mealtimes which emphasizes the importance of optimal eating habits (74). More research is required to determine ideal meal timing and dietary habits for circadian and CM health (25).

In addition to meal timing, the importance of intervals between meals has also been widely discussed. Research data from animal studies has provided evidence that confining daily food intake to 6 to 10 hours and fasting for the remaining hours has beneficial effects on metabolism, even when consuming high-calorie food (49). This led to introduction of the “time-restricted eating (TRE)” concept into human dietary practices. It is a specific form of the more general “intermittent fasting” and involves alternating fasting and normal eating times during specific periods within a day or week (10).

When following TRE schedules, people limit their daily eating window to four to ten hours, without making any effort to limit their calorie or dietary intake.

This method has been shown to increase longevity and health in male mice without affecting the usual diet or daily calorie intake (75). A recent study (76) presented groundbreaking evidence that, in contrast to simple calorie restriction (10% life extension), the positive effects of TRE on health and longevity in mice are greatest (35% life extension) when the feeding interval coincides with the natural active phase of the animal. Time-restricted eating (TRE) has also been suggested for humans as a viable method to restore the rhythmicity of the metabolic pathways that have been disrupted by circadian misalignment.

In one study, IS was enhanced when the eating window was restricted from 7am to 3pm (77). Early TRE significantly improved body weight, waist circumference, beta cell function, and blood pressure in men with prediabetes (78).In another study, obese patients put on an isocaloric early time-restricted eating schedule (e.g., a 6-hour eating window with dinner no later than 3pm) showed decreases in insulin resistance that were much greater than those observed in participants with a 12 hour feeding window (61). Although the exact process by which TRE affects health is not fully understood, a logical explanation could be the increase in robustness of both the rhythmicity of the clock as well as the downstream pathways in metabolic tissue.

Innovative clinical trials have so far presented mixed results (79–81). Further research is required to compare different TRE schedules and their effects in humans (16:8 vs. 14:10; fasting window: eating window). Furthermore, there are few studies that focus on determining the most ideal fasting window and TRE timing (early vs. late) for improvement of health. One major drawback of these human studies is their inability to discern between the effects of TRE and calorie restriction. Although numerous TRE studies have demonstrated some benefits in people with metabolic disorders, it is crucial that further research also include participants who are healthy and do not have weight issues. Moreover, it is important to investigate additional variables, such as long-term adherence to TRE, quality of the diet while on TRE, and the social aspects of adjustment to the TRE lifestyle.

**Figure 4:**
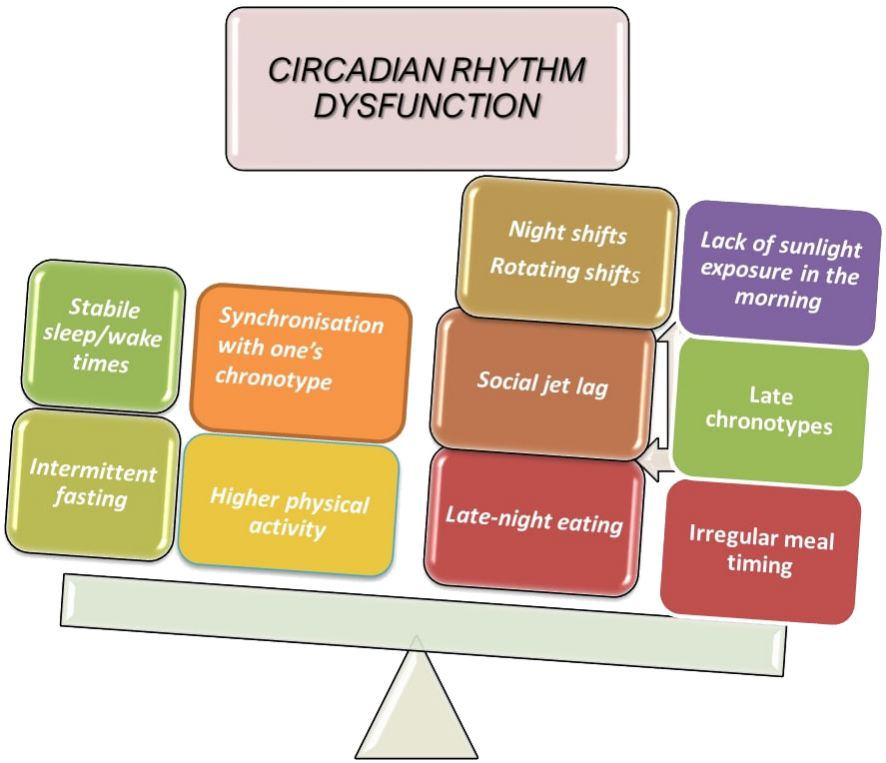
Factors contributing to circadian disruption.

Shift work integrates most of the factors that are known to affect the circadian system (**Figure 4**). In night shift workers, exposure to artificial light could disrupt the central circadian clock, while late night eating disrupts certain peripheral clocks, and this may lead to internal desynchrony. There are, however, additional non-circadian mechanisms which indicate that unhealthy eating habits, insufficient sleep, and decreased PA may contribute to aspects of health problems related to shift work (82). Shift workers are therefore an ideal population to focus on when studying biological and social rhythm disturbances (i.e., the regularity with which one participates in social activities during the week). Working night shifts, particularly when they are part of a rotating shift schedule, is linked to an increased risk of developing T2DM (33). The frequency of night shifts is important because, even after controlling for risk factors and night shift length over the course of a lifetime, higher numbers of average monthly night shifts have been linked to an increase in the prevalence of diabetes (83). It is evident that shift work may lead to irregular meal schedules (i.e., random eating times) and skipping meals, which may have an impact on the hormones that regulate appetite (ghrelin, leptin, neuropeptide Y, and peptide YY) (84). Another condition commonly seen among shift workers is poor sleep, which could partially contribute to their higher risk of developing CVD. Some research has revealed that both shift work and non-shift nurses experience poor sleep quality (85).

A study involving 26 healthy adults was carried out to investigate the effects of sleep restriction on insulin resistance. IS and markers of inflammation were compared in healthy adults under conditions of circadian alignment against misalignment (determined as shift work) with daily sleep duration remaining the same (86). The participants in the above-mentioned study were subjected to either 5 h of sleep restriction with set nocturnal bedtimes (circadian alignment) or an 8.5 h bedtime delay (circadian misalignment). Both interventions comprised 3 inpatient days with a sleep duration of 10 hours, followed by 8 inpatient days with a sleep restriction to 5 hours with fixed nocturnal bedtimes (circadian adjustment) or with bedtimes delayed by 8.5 hours on 4 of the 8 days (circadian misalignment). In both the aligned and misaligned conditions, the daily total sleep time during the intervention was almost the same. After sleep restriction, IS dramatically dropped in both groups without a corresponding rise in insulin secretion, and inflammation went up. When compared to male participants who adhered to regular nocturnal bedtimes, the reduction in IS and the increase in inflammation were both doubled in those exposed to circadian misalignment. In conclusion, inadequate circadian rhythm adjustment in the context of shift work may increase the risk of diabetes and inflammation, while sleep duration is a separate, non-affecting factor in healthy subjects.

### 2.4 Shift work and intestinal microbiota

Over the course of a day, the intestinal microbiota in both humans and mice displays diurnal oscillations that are influenced by eating rhythms, resulting in time-specific compositional and functional profiles (87). Dysbiosis and abnormal microbiota diurnal fluctuations are caused by disruption of the host molecular clock components or induction of circadian desynchrony (jet lag), and are primarily caused by poor eating rhythmicity. When feces are transplanted into germ-free mice, the jet lag-induced dysbiosis in humans and mice leads to glucose intolerance and obesity (87). Collectively, these results demonstrate coordinated diurnal rhythmicity in meta-organisms and suggest a microbiome-dependent mechanism for common metabolic disorders in people with aberrant circadian rhythms, such as those observed in frequent flyers.

A recent study on the microbiomes of ten male security workers who worked both daily and night shifts revealed that the gut microbiota of those who worked night shifts was altered in favor of “obesogenic” bacteria due to rotational day and night shift work (82). This finding raises concerns that the altered gut microbiota caused by shift work may, at minimum, partly account for the higher risk of gastrointestinal disorders and metabolic syndrome. This was corroborated by research showing that the disruption of the microbiome caused by circadian misalignment can also play a role in the development of insulin resistance. The study involved transferring feces from “jet-lagged” humans with a disturbed circadian system into the gut of germ-free mice, which decreased the mice’s ability to tolerate glucose (88).

Mortas et al. found out that abundances of *Bacteroidetes* were reduced and those of *Actinobacteria* and *Firmicutes* increased when working the night compared to day shift. Faecalibacterium abundance was found to be a biomarker of the day shift work. Dorea longicatena and Dorea formicigenerans were significantly more abundant in individuals when working the night shift. Rotational day and night shift work causes circadian rhythm disturbance with an associated alteration in the abundances of gut microbiota, leading to the concern that such induced alteration of gut microbiota may at least partially contribute to an increased risk of future metabolic syndrome and gastrointestinal pathology (82).

Based on the data summarized about shift work above, there is no doubt that it represents a significant risk of developing metabolic disorders and the problem is all the more pressing in light of the rise in shift work that has followed industrialization. Given the increasing prevalence of T2DM, it is imperative to ascertain which aspects of shift work schedules may pose the greatest disruptions, and for whom. This will facilitate the development of focused primary and secondary prevention strategies, which in turn may contribute to a reduction in the societal and financial costs associated with disease. Additionally, more studies need to be done to confirm microbiome-circadian rhythm-metabolic pathology associations because the result can have outcome in targeting some compensation strategies to reverse negative impact of shift work on health.

## 3 Future research

The above-mentioned results suggest that researching the relationship between circadian disruption and metabolic and cardiovascular health in population-based studies is a worthwhile endeavor (26). Despite being carefully planned to test cause and effect, experimental studies are typically conducted over a brief period of time. Since humans are typically exposed to risk factors over longer periods of time in real life, more research is required to determine which factors are the strongest for developing diseases over longer time periods.

The results of studies on the effects of nocturnal night light exposure, sleep disturbance and deprivation, shift work, jet lag, late chronotype and other factors support the hypothesis that disruption of the circadian system contributes to the development of insulin resistance in humans due to impaired glucose tolerance.

The need for more tight integration of the research fields on sleep and CM functions seems obvious because their disturbances represent comorbidity of many disorders. Obstructive sleep apnea (OSA) is example of such disorder. OSA has been shown to be a separate risk factor for cardiovascular morbidity and mortality, as well as an increased risk of hypertension, stroke, acute coronary syndrome, and arrhythmias. The association between OSA and T2DM has been well characterized: on the one hand, OSA can contribute to increased insulin resistance or glucose intolerance; on the other hand, diabetes may worsen sleep-disordered breathing because of autonomic neuropathy. Insulin resistance can also be a predictor for the development of OSA. Moreover, the association between OSA and insulin resistance is probably bidirectional (89). Surprisingly, OSA can also occur in people with type 1 diabetes mellitus (T1DM) who are not obese, despite the fact that it is more commonly associated with T2DM patients (with a prevalence of up to 50%). It has been shown that during sleep, autonomic reactions to hypoglycemia are diminished, especially in individuals with T1DM. Therefore, one potential mechanism of OSA in people with T1DM is neuromuscular dysfunction of the upper airway dilator muscle, which can be impeded by upper airway neuropathy (90). Moreover, a pathological oximetry was linked to advanced age, a longer course of the illness, and a higher incidence of retinopathy (91). Nevertheless, OSA definitely needs more research in the field of CM, which could be very useful for improving the treatment methods of this diagnosis (92).

To determine the ideal meal times and dietary habits for circadian and CM health, more research is required. Timing of eating could be viewed as a novel approach for the diagnosis, prevention, and treatment of T2DM in clinical practice, particularly in vulnerable populations such as shift workers and late-night eaters, who together account for a sizable portion of our society (30). Dietary evaluation is difficult, but techniques that take into account timing of intake—especially in relation to bed and wake times—are essential for this field. Meal and sleep timing is possible with the Automated Self-Administered 24-Hour Dietary Assessment Tool (ASA24), for instance (93).

New molecules targeting the molecular clock by modulating specific clock gene expression have been newly explored as promising targets for improving the circadian regulation such as nobiletin or REV-ERB - nuclear receptor subfamily 1 group D member 1 (NR1D1)) agonists (94). REV-ERB plays an important role in regulation of the circadian clock and it also takes part in several physiological processes, including metabolic pathways and immunity (83).

The new technologies can be employed to determine the underlying mechanism of misalignment between internal circadian rhythmicity and externally imposed behavioral schedules. For example, time-resolved metabolomics has been used as a useful tool. A study published in 2018 found that after simulated shift work, traditional markers of the circadian clock in the SCN (melatonin, cortisol, PER3 gene expression) remained relatively stable but rhythms in many plasma metabolites circulating with 24-hour rhythmicity showed complete reversal or lost rhythms (94). Detailed characterization of the rhythmic metabolite profiles may provide insight into the underlying mechanisms linking shift work and metabolic disorders and help to explore the bio-behavioral factors that orchestrate them (95–97). Additionally, the biomarkers of circadian phase can help to optimize behavioral strategies or possible pharmacological interventions to prevent metabolic disruption in humans in the future.

Finding biological markers to objectively assess the existence of circadian rhythm disruptors in clinical practice is undoubtedly one of the methodological issues that needs to be taken into account for future research. By doing this, the evidence supporting a link between circadian disruption and CM disorders would be more accurate and of higher quality.

In conclusion, it is imperative to investigate optimal compensating mechanisms for shift work, with a particular focus on ways to avoid potential metabolic complications associated with this work schedule. In the future, further clinical studies will be needed to deeply investigate the clinical utility of the current understanding of the regulation of the circadian clock of IS. There is a need to conduct research to see if any of these adjustments advanced or stabilized bedtimes, chronotype-appropriate exercise regimens, and customized meal plans—may be beneficial. This comprises studies to ascertain the efficacy of the intervention as well as implementation studies to evaluate the intervention’s feasibility acceptability in real-world settings. It is crucial that these implementation studies be carried out across a range of demographic subgroups, such as ages, genders, and races and ethnicities, given the significant influence of sociocultural factors on behavior. In conclusion, these upcoming research endeavors will facilitate a more comprehension of circadian disruptors concerning cardio-metabolic disorders and facilitate the identification of efficacious strategies for intervention.

## Author contributions

NM: Writing – original draft, Visualization, Validation, Resources, Methodology, Data curation, Conceptualization, Writing – review & editing. MS: Visualization, Resources, Writing – review & editing. AS: Writing – review & editing, Validation, Supervision, Resources. MD: Supervision, Project administration, Methodology, Funding acquisition, Conceptualization, Writing – review & editing.

## References

[1] Takahashi JS. Transcriptional architecture of the mammalian circadian clock. Nat Rev Genet. (2017) 18:164–79. doi: 10.1038/nrg.2016.150 PMC5501165 27990019

[2] Patke A Young MW Axelrod S. Molecular mechanisms and physiological importance of circadian rhythms. Nat Rev Mol Cell Biol. (2020) 21:67–84. doi: 10.1038/s41580-019-0179-2 31768006

[3] Welsh DK Takahashi JS Kay SA. Suprachiasmatic nucleus: cell autonomy and network properties. Annu Rev Physiol. (2010) 72:551–77. doi: 10.1146/annurev-physiol-021909-135919 PMC3758475 20148688

[4] Damiola F Le Minh N Preitner N Kornmann B Fleury-Olela F Schibler U. Restricted feeding uncouples circadian oscillators in peripheral tissues from the central pacemaker in the suprachiasmatic nucleus. Genes Dev. (2000) 14:2950–61. doi: 10.1101/gad.183500 PMC317100 11114885

[5] Aschoff J Fatranská M Giedke H Doerr P Stamm D Wisser H. Human circadian rhythms in continuous darkness: entrainment by social cues. Science. (1971) 171:213–5. doi: 10.1126/science.171.3967.213 5538832

[6] Colelli DR Cruz Dela GR Kendzerska T Murray BJ Boulos MI. Impact of sleep chronotype on in-laboratory polysomnography parameters. J Sleep Res. (2023) 32:e13922. doi: 10.1111/jsr.13922 37150591

[7] Fabbri M Beracci A Martoni M Meneo D Tonetti L Natale V. Measuring subjective sleep quality: A review. Int J Environ Res Public Health. (2021) 18. doi: 10.3390/ijerph18031082 PMC7908437 33530453

[8] Bradley J O'Neill B Kent L Hulzebos EH Arets B Hebestreit H. Physical activity assessment in cystic fibrosis: A position statement. J Cyst Fibros. (2015) 14:e25–32. doi: 10.1016/j.jcf.2015.05.011 26219990

[9] Telford O Diamantidis CJ Bosworth HB Patel UD Davenport CA Oakes MM. The relationship between Pittsburgh Sleep Quality Index subscales and diabetes control. Chronic Illn. (2019) 15:210–9. doi: 10.1177/1742395318759587 PMC7187808 29466873

[10] Patterson RE Laughlin GA LaCroix AZ Hartman SJ Natarajan L Senger CM. Intermittent fasting and human metabolic health. J Acad Nutr Diet. (2015) 115:1203–12. doi: 10.1016/j.jand.2015.02.018 PMC4516560 25857868

[11] Lunsford-Avery JR Engelhard MM Navar AM. Validation of the sleep regularity index in older adults and associations with cardiometabolic risk. Sci Rep. (2018) 8:14158. doi: 10.1038/s41598-018-32402-5 30242174 PMC6154967

[12] Kuhl W. History of clinical research on the sleep apnea syndrome. The early days of polysomnography. Respiration. (1997) 64 Suppl 1:5–10. doi: 10.1159/000196728 9380961

[13] Smith MT McCrae CS Cheung J Martin JL Harrod CG Heald JL. Use of actigraphy for the evaluation of sleep disorders and circadian rhythm sleep-wake disorders: an american academy of sleep medicine clinical practice guideline. J Clin Sleep Med. (2018) 14:1231–7. doi: 10.5664/jcsm.7230 PMC6040807 29991437

[14] Weissova K Bartoš A Sládek M Nováková M Sumová A. Moderate changes in the circadian system of alzheimer’s disease patients detected in their home environment. PloS One. (2016) 11:e0146200. doi: 10.1371/journal.pone.0146200 26727258 PMC4701009

[15] Matricciani L Dumuid D Paquet C Fraysse F Wang Y Baur LA. Sleep and cardiometabolic health in children and adults: examining sleep as a component of the 24-h day. Sleep Med. (2021) 78:63–74. doi: 10.1016/j.sleep.2020.12.001 33387878

[16] Chinoy ED Cuellar JA Huwa KE Jameson JT Watson CH Bessman SC. Performance of seven consumer sleep-tracking devices compared with polysomnography. Sleep. (2021) 44. doi: 10.1093/sleep/zsaa291 PMC8120339 33378539

[17] Lee HA Lee HJ Moon JH Lee T Kim MG In H. Comparison of wearable activity tracker with actigraphy for sleep evaluation and circadian rest-activity rhythm measurement in healthy young adults. Psychiatry Investig. (2017) 14:179–85. doi: 10.4306/pi.2017.14.2.179 PMC5355016 28326116

[18] Asgari Mehrabadi M Azimi I Sarhaddi F Axelin A Niela-Vilén H Myllyntausta S. Sleep tracking of a commercially available smart ring and smartwatch against medical-grade actigraphy in everyday settings: instrument validation study. JMIR Mhealth Uhealth. (2020) 8:e20465. doi: 10.2196/20465 33038869 PMC7669442

[19] Rutters F Nefs G. Sleep and circadian rhythm disturbances in diabetes: A narrative review. Diabetes Metab Syndr Obes. (2022) 15:3627–37. doi: 10.2147/DMSO.S354026 PMC9694979 36439294

[20] Sladek M Kudrnáčová Röschová M Adámková V Hamplová D Sumová A. Chronotype assessment via a large scale socio-demographic survey favours yearlong Standard time over Daylight Saving Time in central Europe. Sci Rep. (2020) 10:1419. doi: 10.1038/s41598-020-58413-9 31996761 PMC6989656

[21] Roenneberg T Wirz-Justice A Merrow M. Life between clocks: daily temporal patterns of human chronotypes. J Biol Rhythms. (2003) 18:80–90. doi: 10.1177/0748730402239679 12568247

[22] Pagani L Semenova EA Moriggi E Revell VL Hack LM Lockley SW. The physiological period length of the human circadian clock in vivo is directly proportional to period in human fibroblasts. PloS One. (2010) 5:e13376. doi: 10.1371/journal.pone.0013376 21042402 PMC2958564

[23] Roenneberg T Kuehnle T Juda M Kantermann T Allebrandt K Gordijn M. Epidemiology of the human circadian clock. Sleep Med Rev. (2007) 11:429–38. doi: 10.1016/j.smrv.2007.07.005 17936039

[24] Barclay NL Eley TC Buysse DJ Archer SN Gregory AM. Diurnal preference and sleep quality: same genes? A study of young adult twins. Chronobiol Int. (2010) 27:278–96. doi: 10.3109/07420521003663801 20370470

[25] Roenneberg T Kuehnle T Pramstaller PP Ricken J Havel M Guth A. A marker for the end of adolescence. Curr Biol. (2004) 14:R1038–9. doi: 10.1016/j.cub.2004.11.039 15620633

[26] Sladek M Klusáček J Hamplová D Sumová A. Population-representative study reveals cardiovascular and metabolic disease biomarkers associated with misaligned sleep schedules. Sleep. (2023) 46. doi: 10.1093/sleep/zsad037 PMC10262187 36827078

[27] Ghotbi N Pilz LK Winnebeck EC Vetter C Zerbini G Lenssen D. The microMCTQ: an ultra-short version of the munich chronoType questionnaire. J Biol Rhythms. (2020) 35:98–110. doi: 10.1177/0748730419886986 31791166

[28] Martinez-Nicolas A Martinez-Madrid MJ Almaida-Pagan PF Bonmati-Carrion MA Madrid JA Rol MA. Assessing chronotypes by ambulatory circadian monitoring. Front Physiol. (2019) 10:1396. doi: 10.3389/fphys.2019.01396 31824327 PMC6879660

[29] Dubocovich ML. Melatonin receptors: role on sleep and circadian rhythm regulation. Sleep Med. (2007) 8 Suppl 3:34–42. doi: 10.1016/j.sleep.2007.10.007 18032103

[30] Garaulet M Qian J Florez JC Arendt J Saxena R Scheer FAJL. Melatonin effects on glucose metabolism: time to unlock the controversy. Trends Endocrinol Metab. (2020) 31:192–204. doi: 10.1016/j.tem.2019.11.011 31901302 PMC7349733

[31] Gnocchi D Bruscalupi G. Circadian rhythms and hormonal homeostasis: pathophysiological implications. Biol (Basel). (2017) 6. doi: 10.3390/biology6010010 PMC5372003 28165421

[32] Baldanzi G Hammar U Fall T Lindberg E Lind L Elmståhl S. Evening chronotype is associated with elevated biomarkers of cardiometabolic risk in the EpiHealth cohort: a cross-sectional study. Sleep. (2022) 45. doi: 10.1093/sleep/zsab226 PMC8842133 34480568

[33] Knutson KL Spiegel K Penev P Van Cauter E. The metabolic consequences of sleep deprivation. Sleep Med Rev. (2007) 11:163–78. doi: 10.1016/j.smrv.2007.01.002 PMC1991337 17442599

[34] Docimo A Verde L Barrea L Vetrani C Memoli P Accardo G. Type 2 diabetes: also a “Clock matter”? Nutrients. (2023) 15. doi: 10.3390/nu15061427 PMC10059837 36986157

[35] Finn L Young T Palta M Fryback DG. Sleep-disordered breathing and self-reported general health status in the Wisconsin Sleep Cohort Study. Sleep. (1998) 21:701–6. doi: 10.1371/journal.pone.0133761 11286346

[36] Merikanto I Lahti T Puolijoki H Vanhala M Peltonen M Laatikainen T. Associations of chronotype and sleep with cardiovascular diseases and type 2 diabetes. Chronobiol Int. (2013) 30:470–7. doi: 10.3109/07420528.2012.741171 23281716

[37] Vetter C Devore EE Ramin CA Speizer FE Willett WC Schernhammer ES. Mismatch of sleep and work timing and risk of type 2 diabetes. Diabetes Care. (2015) 38:1707–13. doi: 10.2337/dc15-0302 PMC4542269 26109502

[38] Santhi N Lazar AS McCabe PJ Lo JC Groeger JA Dijk DJ. Sex differences in the circadian regulation of sleep and waking cognition in humans. Proc Natl Acad Sci U.S.A. (2016) 113:E2730–9. doi: 10.1073/pnas.1521637113 PMC4868418 27091961

[39] Green R Polotsky AJ Wildman RP McGinn AP Lin J Derby C. Menopausal symptoms within a Hispanic cohort: SWAN, the Study of Women’s Health Across the Nation. Climacteric. (2010) 13:376–84. doi: 10.3109/13697130903528272 PMC3268678 20136411

[40] Peplonska B Bukowska A Sobala W. Association of rotating night shift work with BMI and abdominal obesity among nurses and midwives. PloS One. (2015) 10:e0133761. doi: 10.1371/journal.pone.0133761 26196859 PMC4511417

[41] Cho K Ennaceur A Cole JC Suh CK. Chronic jet lag produces cognitive deficits. J Neurosci. (2000) 20:RC66. doi: 10.1523/JNEUROSCI.20-06-j0005.2000 10704520 PMC6772481

[42] Haufe A Leeners B. Sleep disturbances across a woman’s lifespan: what is the role of reproductive hormones? J Endocr Soc. (2023) 7:bvad036. doi: 10.1210/jendso/bvad036 37091307 PMC10117379

[43] Sudlow C Gallacher J Allen N Beral V Burton P Danesh J. UK biobank: an open access resource for identifying the causes of a wide range of complex diseases of middle and old age. PloS Med. (2015) 12:e1001779. doi: 10.1371/journal.pmed.1001779 25826379 PMC4380465

[44] Vetter C Dashti HS Lane JM Anderson SG Schernhammer ES Rutter MK. Night shift work, genetic risk, and type 2 diabetes in the UK biobank. Diabetes Care. (2018) 41:762–9. doi: 10.2337/dc17-1933 PMC5860836 29440150

[45] Wang M Zhou T Li X Ma H Liang Z Fonseca VA. Baseline vitamin D status, sleep patterns, and the risk of incident type 2 diabetes in data from the UK biobank study. Diabetes Care. (2020) 43:2776–84. doi: 10.2337/dc20-1109 PMC7576418 32847829

[46] Scott RA Scott LJ Mägi R Marullo L Gaulton KJ Kaakinen M. An expanded genome-wide association study of type 2 diabetes in europeans. Diabetes. (2017) 66:2888–902. doi: 10.2337/db16-1253 PMC5652602 28566273

[47] Poggiogalle E Jamshed H Peterson CM. Circadian regulation of glucose, lipid, and energy metabolism in humans. Metabolism. (2018) 84:11–27. doi: 10.1016/j.metabol.2017.11.017 29195759 PMC5995632

[48] Saad A Dalla Man C Nandy DK Levine JA Bharucha AE Rizza RA. Diurnal pattern to insulin secretion and insulin action in healthy individuals. Diabetes. (2012) 61:2691–700. doi: 10.2337/db11-1478 PMC3478548 22751690

[49] Panda S. Circadian physiology of metabolism. Science. (2016) 354:1008–15. doi: 10.1126/science.aah4967 PMC7261592 27885007

[50] Scheer FA Hu K Evoniuk H Kelly EE Malhotra A Hilton MF. Impact of the human circadian system, exercise, and their interaction on cardiovascular function. Proc Natl Acad Sci U.S.A. (2010) 107:20541–6. doi: 10.1073/pnas.1006749107 PMC2996667 21059915

[51] Westerterp-Plantenga MS Drummen M Tischmann L Swindell N Stratton G Raben A. Circadian rhythm parameters and physical activity associated with cardiometabolic risk factors in the PREVIEW lifestyle study. Obes (Silver Spring). (2023) 31:744–56. doi: 10.1002/oby.23670 36782388

[52] Estrella MA Du J Chen L Rath S Prangley E Chitrakar A. The metabolites NADP(+) and NADPH are the targets of the circadian protein Nocturnin (Curled). Nat Commun. (2019) 10:2367. doi: 10.1038/s41467-019-10125-z 31147539 PMC6542800

[53] Green CB Douris N Kojima S Strayer CA Fogerty J Lourim D. Loss of Nocturnin, a circadian deadenylase, confers resistance to hepatic steatosis and diet-induced obesity. Proc Natl Acad Sci U.S.A. (2007) 104:9888–93. doi: 10.1073/pnas.0702448104 PMC1871564 17517647

[54] Eckel-Mahan KL Patel VR de Mateo S Orozco-Solis R Ceglia NJ Sahar S. Reprogramming of the circadian clock by nutritional challenge. Cell. (2013) 155:1464–78. doi: 10.1016/j.cell.2013.11.034 PMC4573395 24360271

[55] Gnocchi D Custodero C Sabbà C Mazzocca A. Circadian rhythms: a possible new player in non-alcoholic fatty liver disease pathophysiology. J Mol Med (Berl). (2019) 97:741–59. doi: 10.1007/s00109-019-01780-2 30953079

[56] Gnocchi D Pedrelli M Hurt-Camejo E Parini P. Lipids around the clock: focus on circadian rhythms and lipid metabolism. Biol (Basel). (2015) 4:104–32. doi: 10.3390/biology4010104 PMC4381220 25665169

[57] Yanagihara H Ando H Hayashi Y Obi Y Fujimura A. High-fat feeding exerts minimal effects on rhythmic mRNA expression of clock genes in mouse peripheral tissues. Chronobiol Int. (2006) 23:905–14. doi: 10.1080/07420520600827103 17050208

[58] Kubota Y Evenson KR Maclehose RF Roetker NS Joshu CE Folsom AR. Physical activity and lifetime risk of cardiovascular disease and cancer. Med Sci Sports Exerc. (2017) 49:1599–605. doi: 10.1249/MSS.0000000000001274 PMC5511058 28350711

[59] Rubio-Sastre P Scheer FA Gómez-Abellán P Madrid JA Garaulet M. Acute melatonin administration in humans impairs glucose tolerance in both the morning and evening. Sleep. (2014) 37:1715–9. doi: 10.5665/sleep.4088 PMC4173928 25197811

[60] Jarrin DC Ivers H Lamy M Chen IY Harvey AG Morin CM. Cardiovascular autonomic dysfunction in insomnia patients with objective short sleep duration. J Sleep Res. (2018) 27:e12663. doi: 10.1111/jsr.12663 29493063 PMC5992004

[61] Gauthier P Desir C Plombas M Joffray E Benhamou PY Borel AL. Impact of sleep and physical activity habits on real-life glycaemic variability in patients with type 2 diabetes. J Sleep Res. (2023) 32:e13799. doi: 10.1111/jsr.13799 36495012

[62] Tranel HR Schroder EA England J Black WS Bush H Hughes ME. Physical activity, and not fat mass is a primary predictor of circadian parameters in young men. Chronobiol Int. (2015) 32:832–41. doi: 10.3109/07420528.2015.1043011 PMC4550083 26101893

[63] Menek MY Budak M. Effect of exercises according to the circadian rhythm in type 2 diabetes: Parallel-group, single-blind, crossover study. Nutr Metab Cardiovasc Dis. (2022) 32:1742–52. doi: 10.1016/j.numecd.2022.04.017 35606229

[64] van Moorsel D Hansen J Havekes B Scheer FAJL Jörgensen JA Hoeks J. Demonstration of a day-night rhythm in human skeletal muscle oxidative capacity. Mol Metab. (2016) 5:635–45. doi: 10.1016/j.molmet.2016.06.012 PMC5021670 27656401

[65] Bruce CR Anderson MJ Carey AL Newman DG Bonen A Kriketos AD. Muscle oxidative capacity is a better predictor of insulin sensitivity than lipid status. J Clin Endocrinol Metab. (2003) 88:5444–51. doi: 10.1210/jc.2003-030791 14602787

[66] Pickel L Sung HK. Feeding rhythms and the circadian regulation of metabolism. Front Nutr. (2020) 7:39. doi: 10.3389/fnut.2020.00039 32363197 PMC7182033

[67] Dickmeis T Weger BD Weger M. The circadian clock and glucocorticoids–interactions across many time scales. Mol Cell Endocrinol. (2013) 380:2–15. doi: 10.1016/j.mce.2013.05.012 23707790

[68] Dijk DJ Duffy JF Silva EJ Shanahan TL Boivin DB Czeisler CA. Amplitude reduction and phase shifts of melatonin, cortisol and other circadian rhythms after a gradual advance of sleep and light exposure in humans. PloS One. (2012) 7:e30037. doi: 10.1371/journal.pone.0030037 22363414 PMC3281823

[69] Doane LD Kremen WS Eaves LJ Eisen SA Hauger R Hellhammer D. Associations between jet lag and cortisol diurnal rhythms after domestic travel. Health Psychol. (2010) 29:117–23. doi: 10.1037/a0017865 PMC3089060 20230083

[70] Leproult R Holmback U Van Cauter E. Circadian misalignment augments markers of insulin resistance and inflammation, independently of sleep loss. Diabetes. (2014) 63:1860–9. doi: 10.2337/db13-1546 PMC4030107 24458353

[71] Schiavo-Cardozo D Lima MM Pareja JC Geloneze B. Appetite-regulating hormones from the upper gut: disrupted control of xenin and ghrelin in night workers. Clin Endocrinol (Oxf). (2013) 79:807–11. doi: 10.2337/db13-1546 23199168

[72] Mosavat M Mirsanjari M Arabiat D Smyth A Whitehead L. The role of sleep curtailment on leptin levels in obesity and diabetes mellitus. Obes Facts. (2021) 14:214–21. doi: 10.1159/000514095 PMC8138234 33756469

[73] Sakai R Hashimoto Y Ushigome E Miki A Okamura T Matsugasumi M. Late-night-dinner is associated with poor glycemic control in people with type 2 diabetes: The KAMOGAWA-DM cohort study. Endocr J. (2018) 65:395–402. doi: 10.1507/endocrj.EJ17-0414 29375081

[74] Kelly KP McGuinness OP Buchowski M Hughey JJ Chen H Powers J. Eating breakfast and avoiding late-evening snacking sustains lipid oxidation. PloS Biol. (2020) 18:e3000622. doi: 10.1371/journal.pbio.3000622 32108181 PMC7046182

[75] Chaix A Zarrinpar A Miu P Panda S. Time-restricted feeding is a preventative and therapeutic intervention against diverse nutritional challenges. Cell Metab. (2014) 20:991–1005. doi: 10.1016/j.cmet.2014.11.001 25470547 PMC4255155

[76] Acosta-Rodriguez V Rijo-Ferreira F Izumo M Xu P Wight-Carter M Green CB. Circadian alignment of early onset caloric restriction promotes longevity in male C57BL/6J mice. Science. (2022) 376:1192–202. doi: 10.1126/science.abk0297 PMC9262309 35511946

[77] Odegaard AO Jacobs DR Jr Steffen LM Van Horn L Ludwig DS Pereira MA. Breakfast frequency and development of metabolic risk. Diabetes Care. (2013) 36:3100–6. doi: 10.2337/dc13-0316 PMC3781522 23775814

[78] Sutton EF Beyl R Early KS Cefalu WT Ravussin E Peterson CM. Early time-restricted feeding improves insulin sensitivity, blood pressure, and oxidative stress even without weight loss in men with prediabetes. Cell Metab. (2018) 27:1212–1221 e3. doi: 10.1016/j.cmet.2018.04.010 29754952 PMC5990470

[79] In Het Panhuis W Schönke M Modder M Tom HE Lalai RA Pronk ACM. Time-restricted feeding attenuates hypercholesterolaemia and atherosclerosis development during circadian disturbance in APOE *3-Leiden.CETP mice. EBioMedicine. (2023) 93:104680. doi: 10.1016/j.ebiom.2023.104680 37356205 PMC10320519

[80] Liu D Huang Y Huang C Yang S Wei X Zhang P. Calorie restriction with or without time-restricted eating in weight loss. N Engl J Med. (2022) 386:1495–504. doi: 10.1056/NEJMoa2114833 35443107

[81] Wilkinson MJ Manoogian ENC Zadourian A Lo H Fakhouri S Shoghi A. Ten-hour time-restricted eating reduces weight, blood pressure, and atherogenic lipids in patients with metabolic syndrome. Cell Metab. (2020) 31:92–104 e5. doi: 10.1016/j.cmet.2019.11.004 31813824 PMC6953486

[82] Mortas H Bilici S Karakan T. The circadian disruption of night work alters gut microbiota consistent with elevated risk for future metabolic and gastrointestinal pathology. Chronobiol Int. (2020) 37:1067–81. doi: 10.1080/07420528.2020.1778717 32602753

[83] Yin L Wu N Lazar MA. Nuclear receptor Rev-erbalpha: a heme receptor that coordinates circadian rhythm and metabolism. Nucl Recept Signal. (2010) 8:e001. doi: 10.1621/nrs.08001 20414452 PMC2858265

[84] Chaput JP McHill AW Cox RC Broussard JL Dutil C da Costa BGG. The role of insufficient sleep and circadian misalignment in obesity. Nat Rev Endocrinol. (2023) 19:82–97. doi: 10.1038/s41574-022-00747-7 36280789 PMC9590398

[85] Huang Q Tian C Zeng XT. Poor sleep quality in nurses working or having worked night shifts: A cross-sectional study. Front Neurosci. (2021) 15:638973. doi: 10.3389/fnins.2021.638973 34413721 PMC8369413

[86] Xie Z Sun Y Ye Y Hu D Zhang H He Z. Randomized controlled trial for time-restricted eating in healthy volunteers without obesity. Nat Commun. (2022) 13:1003. doi: 10.1038/s41467-022-28662-5 35194047 PMC8864028

[87] Thaiss CA Zeevi D Levy M Zilberman-Schapira G Suez J Tengeler AC. Transkingdom control of microbiota diurnal oscillations promotes metabolic homeostasis. Cell. (2014) 159:514–29. doi: 10.1016/j.cell.2014.09.048 25417104

[88] Poroyko VA Carreras A Khalyfa A Khalyfa AA Leone V Peris E. Chronic sleep disruption alters gut microbiota, induces systemic and adipose tissue inflammation and insulin resistance in mice. Sci Rep. (2016) 6:35405. doi: 10.1038/srep35405 27739530 PMC5064361

[89] Framnes SN Arble DM. The bidirectional relationship between obstructive sleep apnea and metabolic disease. Front Endocrinol (Lausanne). (2018) 9:440. doi: 10.3389/fendo.2018.00440 30127766 PMC6087747

[90] Manin G Pons A Baltzinger P Moreau F Iamandi C Wilhelm JM. Obstructive sleep apnoea in people with Type 1 diabetes: prevalence and association with micro- and macrovascular complications. Diabetes Med. (2015) 32:90–6. doi: 10.1111/dme.12582 25186832

[91] Borel AL Benhamou PY Baguet JP Halimi S Levy P Mallion JM. High prevalence of obstructive sleep apnoea syndrome in a Type 1 diabetic adult population: a pilot study. Diabetes Med. (2010) 27:1328–9. doi: 10.1111/j.1464-5491.2010.03096.x 20950392

[92] Larcher S Gauchez AS Lablanche S Pépin JL Benhamou PY Borel AL. Impact of sleep behavior on glycemic control in type 1 diabetes: the role of social jetlag. Eur J Endocrinol. (2016) 175:411–9. doi: 10.1530/EJE-16-0188 27530460

[93] Chakradeo P Rasmussen HE Swanson GR Swanson B Fogg LF Bishehsari Fv. Psychometric testing of a food timing questionnaire and food timing screener. Curr Dev Nutr. (2022) 6:nzab148. doi: 10.1093/cdn/nzab148 35198845 PMC8856943

[94] Dose B Yalçin M Dries SPM Relógio A. TimeTeller for timing health: The potential of circadian medicine to improve performance, prevent disease and optimize treatment. Front Digit Health. (2023) 5:1157654. doi: 10.3389/fdgth.2023.1157654 37153516 PMC10155816

[95] Harding BN Skene DJ Espinosa A Middleton B Castaño-Vinyals G Papantoniou K. Metabolic profiling of night shift work - The HORMONIT study. Chronobiol Int. (2022) 39:1508–16. doi: 10.1080/07420528.2022.2131562 PMC10482506 36210507

[96] Isherwood CM Van der Veen DR Johnston JD Skene DJ. Twenty-four-hour rhythmicity of circulating metabolites: effect of body mass and type 2 diabetes. FASEB J. (2017) 31:5557–67. doi: 10.1096/fj.201700323R PMC5690388 28821636

[97] Woelders T Revell VL Middleton B Ackermann K Kayser M Raynaud FI. Machine learning estimation of human body time using metabolomic profiling. Proc Natl Acad Sci U.S.A. (2023) 120:e2212685120. doi: 10.1073/pnas.2212685120 37094145 PMC10161018

